# Supplementary material for: Combination of Cellulose Derivatives and Chitosan-Based Polymers to Investigate the Effect of Permeation Enhancers Added to In Situ Nasal Gels for the Controlled Release of Loratadine and Chlorpheniramine
Source: Polymers (Basel). 2023 Feb 27;15(5):1206. doi: 10.3390/polym15051206 (PMC10006938; doi:10.3390/polym15051206)
Supplement: Supplementary file 1 [file polymers-15-01206-s001.zip › polymers-2143609-supplementary.pdf]

polymers

Chitosan is a strong base owing to the presence of primary amino groups, and becomes a polyelectrolyte when such amino groups become protonated. As a result, chitosan dissolves in acid aqueous mixtures (e.g., aqueous acetic acid).

Chitosan dissolves in various aqueous solutions containing organic and inorganic acids because it is only a basic polymer occurring naturally. Carbonic acid gas (CO_2 gas) is a substance that acts as an acid for dissolving chitosan in water and is naturally removed in the process of preparing formulations.

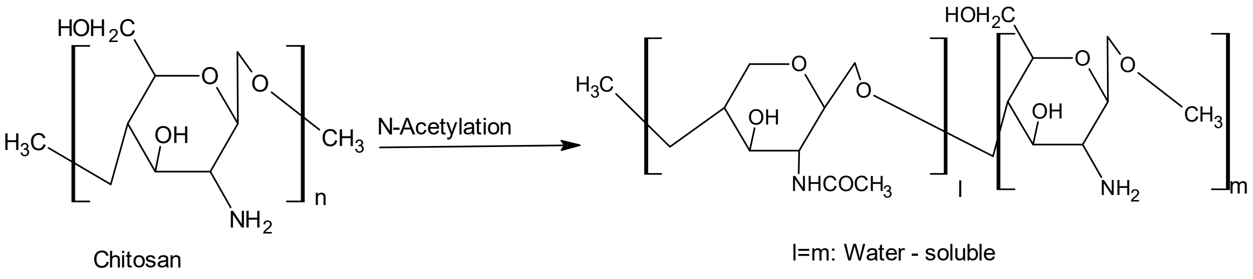

Figure S1: N-Acetylation of chitosan

Chitosan undergoes N acetylation to form water-soluble chitosan; hence, there is a single degree of acetylisation.

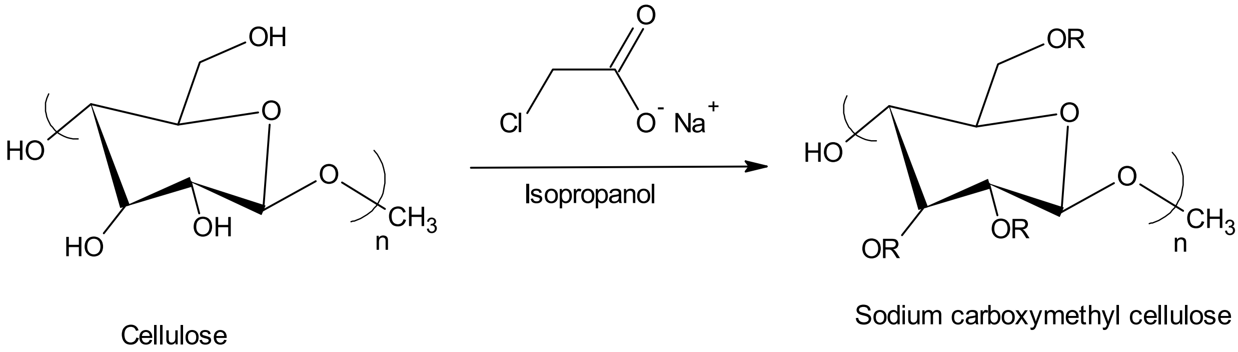

Cellulose derivatives, when treated with isopropanol esterification, occur in the prescence of sodium hydroxide to form sodium carboxy methyl cellulose. Sodium carboxy methyl cellulose has mainly three substitution sites; if the cellulose was not fully substituted, three degrees of substitution reaction should be carried out for completely substituted sodium carboxymethylcellulose.
